# An investigation of the load-velocity relationship between flywheel eccentric and barbell training methods

**DOI:** 10.3389/fpubh.2025.1579291

**Published:** 2025-05-30

**Authors:** Ziwei Zhu, Jiayong Chen, Ruize Sun, Renchen Wang, Jiaxin He, Wenfeng Zhang, Weilong Lin, Duanying Li

**Affiliations:** ^1^Graduate School, Guangzhou Sport University, Guangzhou, China; ^2^Harbin Institute of Information Technology, Harbin, China; ^3^School of Athletic Training, Guangzhou Sport University, Guangzhou, China

**Keywords:** flywheel resistance training, load monitoring, sports performance, digital training, velocity-based training

## Abstract

**Objective:**

Flywheel resistance training (FRT) is a training modality for developing lower limb athletic performance. The relationship between FRT load parameters and barbell squat loading remains ambiguous in practice, resulting in experience-driven load selection during training. Therefore, this study investigates optimal FRT loading for specific training goals (maximal strength, power, muscular endurance) by analyzing concentric velocity at varying barbell 1RM percentages (%1RM), establishes correlations between flywheel load, velocity, and %1RM, and integrates force-velocity profiling to develop evidence-based guidelines for individualized load prescription.

**Methods:**

Thirty-nine participants completed 1RM barbell squats to establish submaximal loads (20–90%1RM). Concentric velocities were monitored via linear-position transducer (Gymaware) for FRT inertial load quantification, with test–retest measurements confirming protocol reliability. Simple and multiple linear regression modeled load-velocity interactions and multivariable relationships, while Pearson’s r and R^2^ quantified correlations and model fit. Predictive equations estimated inertial loads (kg·m^2^), supported by ICC (2, 1) and CV assessments of relative/absolute reliability.

**Results:**

A strong inverse correlation (r = −0.88) and high linearity (R^2^ = 0.78) emerged between rotational inertia and velocity. The multivariate model demonstrated excellent fit (R^2^ = 0.81) and robust correlation (r = 0.90), yielding the predictive equation: y = 0.769–0.846v + 0.002 kg.

**Conclusion:**

The strong linear inertial load-velocity relationship enables individualized load prescription through regression equations incorporating velocity and strength parameters. While FRT demonstrates limited efficacy for developing speed-strength, its longitudinal periodization effects require further investigation. Optimal FRT loading ranges were identified: 40–60%1RM for strength-speed, 60–80%1RM for power development, and 80–100% + 1RM for maximal strength adaptations.

## 1 Introduction

In high-level competitive training, scientifically precise load monitoring not only effectively prevents sports injuries but also significantly enhances athletic performance, serving as an indispensable key component of the training process (1). Studies on load-injury relationships indicate a strong correlation (2, 3), and load monitoring helps coaches avoid prescribing excessively high or low training loads during program design. Training load monitoring can be categorized into internal and external load monitoring. Taking barbell resistance training (RT) as an example, coaches typically design and regulate external training loads based on athletes’ one-repetition maximum (1RM) to achieve predetermined training objectives (4, 5). With the widespread adoption of visualization devices, metrics such as power and velocity have been integrated into resistance training load monitoring, providing guidance for developing precise load regulation strategies (1, 6). Therefore, establishing an effective and targeted training load monitoring system is crucial for improving athletic performance and preventing injuries.

Flywheel resistance training (FRT) is a specialized training modality that involves performing work against the inertial resistance of a rotating flywheel. FRT load is quantified by the moment of inertia (kg·m^2^), with resistance originating from the flywheel’s mass, radius, and pulling velocity during the concentric phase (7). This mechanism enables athletes to generate maximal force during concentric actions while inducing transient eccentric overload (EO) in the subsequent eccentric phase (8, 9). Flywheel inertia discs with varying moments of inertia differentially influence mechanical output: higher inertia may reduce power output but significantly enhance force production (10), whereas lower inertia may benefit individuals with “velocity deficits” (11). Studies have demonstrated that FRT effectively promotes muscle hypertrophy and strength gains (12, 13), attributed to unique neuromuscular adaptation mechanisms elicited by its coupled concentric-eccentric movement patterns (12–14). However, systematic guidelines for FRT load selection remain underdeveloped (15), with current research predominantly focusing on training effects under fixed-load conditions (12, 16). For instance, Davó et al. (17) and Gonzalo-Skok et al. (18) employed loads of 0.025 kg·m^2^ and 0.27 kg·m^2^, respectively, for power development, while Sabido et al. (19) utilized 0.05 kg·m^2^ for maximal strength and power enhancement. Although these studies demonstrated positive outcomes, the fixed-load paradigm may fail to achieve optimal adaptive effects due to inter-individual strength variations (20).

Recent research suggests implementing velocity-based metrics for FRT load intensity monitoring (7, 11), as increased flywheel inertia leads to velocity decrements (11), making velocity tracking a potentially effective monitoring approach. Supporting this perspective, studies have demonstrated that utilizing mean concentric velocity could serve as an effective method for personalized training prescription (16).

Studies have confirmed significant correlations between the application of force-velocity profiling and athletic performance (21). From a biomechanical perspective, the force-velocity theory reveals that increased loading leads to a reduction in peak concentric velocity (22). a principle that has also been validated in FRT contexts (16). Research further indicates that the concentric phase contributes substantially to training outcomes (23). Necessitating equivalent emphasis on concentric velocity monitoring in FRT. This study aims to investigate optimal FRT loading patterns for specific athletic capacity development by analyzing concentric velocities corresponding to different 1RM% in barbell training, with the goal of establishing quantitative relationship models among flywheel load, velocity, and 1RM%. Research hypotheses propose: (a) significant linear relationships exist between FRT inertial load and velocity; (b) multivariate linear regression equations can be established to quantitatively characterize loading parameters; (c) predict the capability development corresponding to FRT under barbell velocity.

## 2 Methods

### 2.1 Experimental approach to the problem

This study utilized a cross-sectional testing design. Participants completed four testing sessions, with a 48-h interval between each session. The 48-h interval was implemented because muscles recover more effectively 48 h after eccentric training (24, 25). Testing was conducted in an indoor strength training facility. Before formal testing, all participants underwent a familiarization session to standardize squat depth and practice FRT procedures, ensuring correct movement execution during testing.

### 2.2 Subjects

The sample size was calculated using G-power 3.1.9.7. The results indicated that for a multivariate linear regression design, with an effect size (ES) of 0.30, an alpha error probability of 0.05, and a power of 0.8, the study should recruit 36 participants. Considering potential sample attrition, an additional 20% was added, resulting in a target recruitment of 43 participants. Initially, a total of 45 individuals were recruited. However, two participants withdrew from the study due to injury, and four participants were excluded due to multiple absences. Ultimately, the study included a total of 39 participants.

All participants were college athletes from the Sports University (Table 1). The athletes were informed about the potential benefits and risks associated with participating in the testing process. They were also informed that they had the right to withdraw from the study at any time and that repeated absences would result in mandatory withdrawal. During the testing period, participants were not allowed to participate in any other experiments. All participants provided informed consent by signing a consent form. This study was approved by the Ethics Committee of Guangzhou Sport University (Approval ID: 2023LCLL-37).

**Table 1:** Basic information of subjects (*n* = 39).

Variable	Mean ± SD
Age (year)	20.28 ± 1.57
Weight (kg)	78.11 ± 9.24
Height (cm)	182.35 ± 8.81
BMI (kg/m^2^)	23.36 ± 1.84
Training years (year)	6.10 ± 2.47
Squat 1RM (kg)	139.48 ± 22.38

### 2.3 Procedure

**Figure 1:**
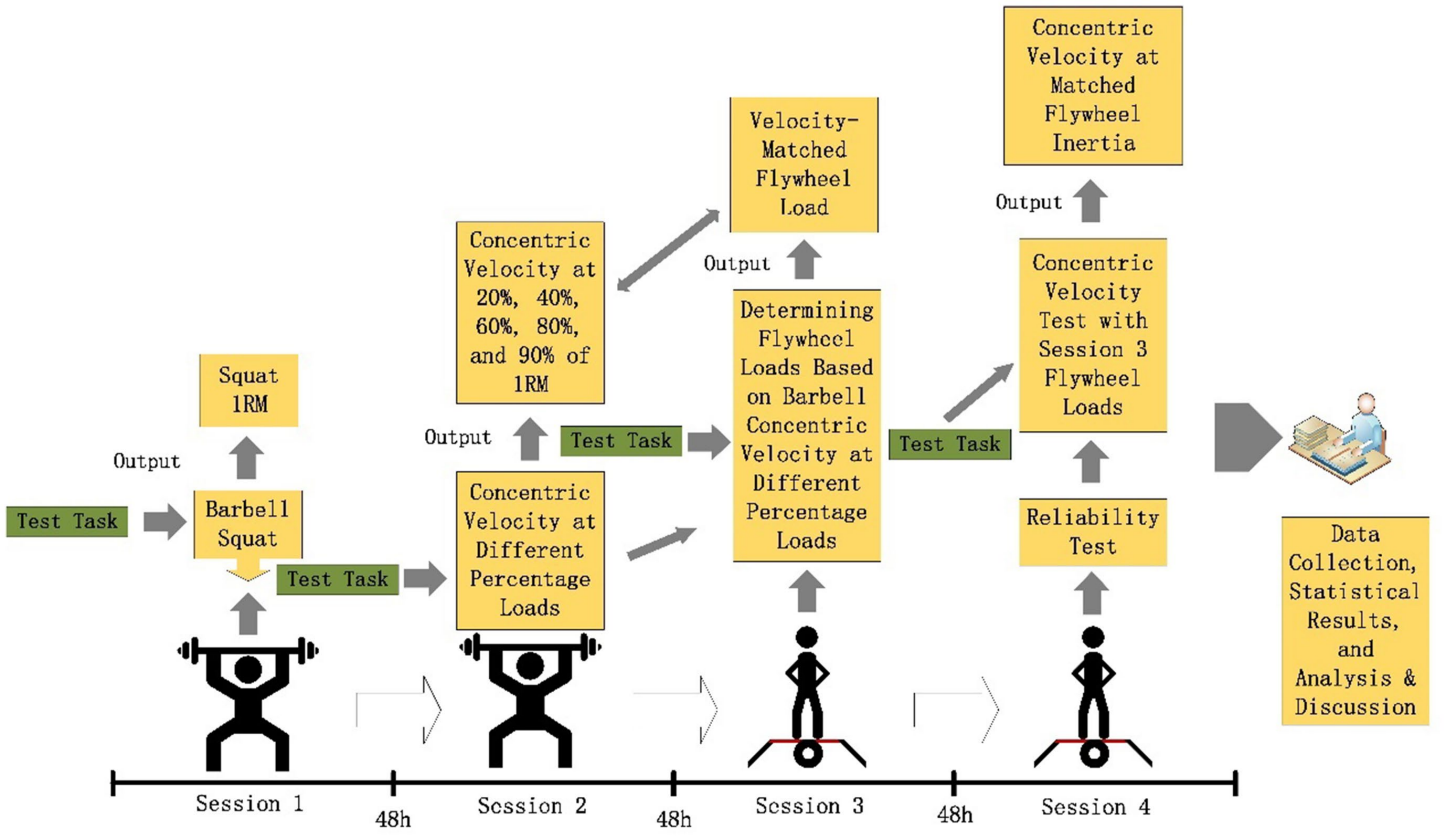
Experimental flowchart.

Prior to testing, participants completed a standardized warm-up protocol involving dynamic stretching (including lunge stretches, knee-to-chest stretches with hip abduction, straight-leg toe touches, heel-walking for 15 meters, and compound dynamic stretches, five repetitions per side) and neuromuscular activation exercises (high-knee marching over 15 meters, lateral shuffles for 5 s followed by sprints, rapid hip rotations for 5 s followed by sprints) to enhance lower limb muscle elasticity and neural activation for improved test validity. Testing comprised four sessions separated by 48-h intervals: Session 1 (1RM back squat), Session 2 (barbell squat velocity at 20, 40, 60, 80, and 90% 1RM), Session 3 (flywheel load determination matching barbell velocities from Session 2), and Session 4 (retesting Session 3 loads to assess reliability via concentric velocity comparison). Baseline anthropometrics (body weight, BMI) were collected during familiarization using Inbody370. All session’s required 48-h recovery, standardized athletic attire, maximal effort during testing, and controlled environmental conditions (temperature: 27.15 ± 1.16°C; humidity: 64.79 ± 6.95%). Test administrators provided uniform verbal encouragement throughout (Figure 1).

### 2.4 Squat 1RM and load-velocity relationship testing

During barbell back squats, participants gripped the barbell with a closed pronated grip in a high-bar position, feet placed slightly wider than shoulder-width. The movement required simultaneous hip and knee flexion while maintaining a fixed torso angle relative to the ground, with knees aligned in the direction of the toes. The eccentric phase concluded when thighs reached parallel to the ground, immediately followed by the concentric phase. Prior to maximal strength testing, an estimated 1RM value was determined for each participant, with permissible deviations in load selection. The protocol proceeded through the following steps: (1) 10 repetitions with an unloaded barbell as warm-up, followed by 2–3 min of rest; (2) subsequent sets involved load increments of approximately 15% of the estimated 1RM (3–5 repetitions per set), with 3–5 min of rest between sets; (3) upon reaching 90% of the estimated 1RM, loads were increased by 5% per attempt (1–2 repetitions per set) with 5-min rest intervals; (4) final attempts at the estimated 1RM: successful lifts were followed by 5 min of rest and a 5% load increase, while failures required 5 min of rest and reattempts, with repeated failures leading to 5 min of rest and a 2.5–5% load reduction. Participants established their 1RM within five attempts (4, 26).

**Figure 2:**
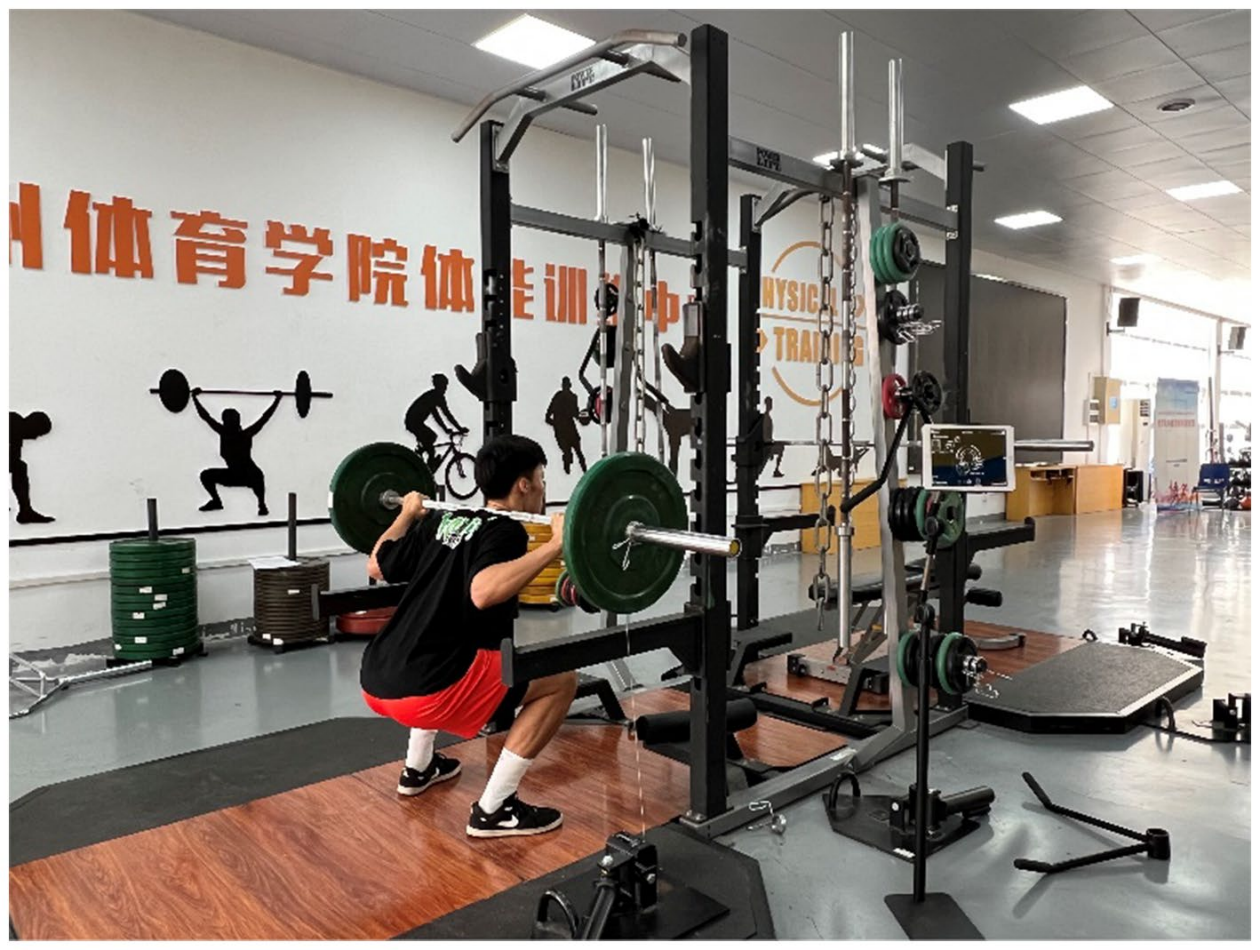
Barbell squats.

During velocity zone testing, a GymAware device (Kinetic Performance Technology, Australia) was attached to one side of the barbell and positioned perpendicular to the ground. Participants performed maximal-effort barbell squats after the researcher issued a “Go” command. Load-Velocity relationship testing across different percentage loads followed the protocol established by Banyard et al. (27) (PV: 20–100%1RM, r = 0.91–0.93; MPV: 20–90%1RM, r = 0.92–0.94; MV: 20–90%1RM, r = 0.94–0.95). Three squats were performed at 20%1RM, 40%1RM, and 60%1RM, with one squat at 80%1RM and 90%1RM; all trials were averaged for final data analysis, with 2–4 min rest between sets (20, 27, 28) (Figure 2).

### 2.5 Flywheel test

**Figure 3:**
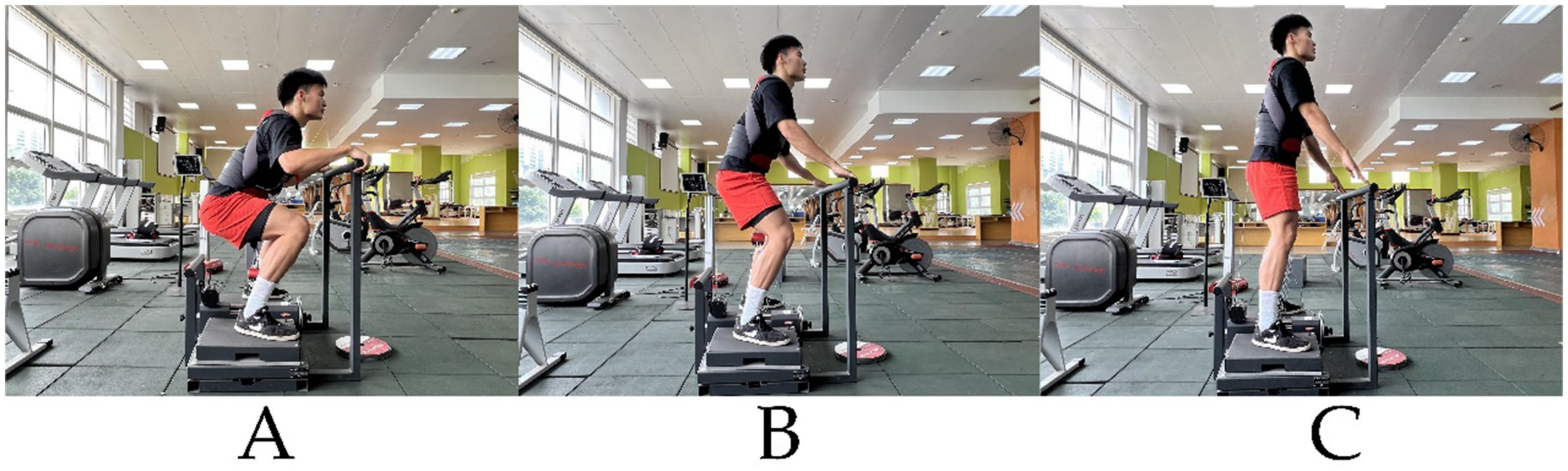
Flywheel Squat. **(A)** Eccentric phase of the squat. **(B)** Concentric phase of the squat. **(C)** Completion of the movement.

The study utilized a flywheel device (D. FULL) from Desmotec (Italy), equipped with seven inertial discs: two small (S, 0.048 kg·m^2^), one medium (M, 0.063 kg·m^2^), two large (L, 0.162 kg·m^2^), and two pro (P, 0.487 kg·m^2^), with moments of inertia calculated as I = m (mass) × r (radius)^2^. Prior to testing, participants adjusted the flywheel strap length in a fully upright stance, while a GymAware device (Kinetic Performance Technology, Australia) was affixed to the rear of a wearable vest using double-sided adhesive tape and positioned vertically beneath the participant. During testing, participants squatted to thigh-parallel ground position, gripped a front support bar, maximally exerted effort during the concentric phase, and controlled resistance during the eccentric return. Flywheel load-velocity relationship testing mirrored barbell protocols with randomized trial orders to prevent fatigue. Five load intensities (20, 40, 60, 80, 90%1RM) were tested: 20–60%1RM involved six squats [first three excluded as warm-up (7), final three averaged]; 80–90%1RM included four squats (last trial recorded). Inertial discs were incrementally added from smallest to largest during load exploration, with 3–5 min intra-set and 4–5 min inter-load rests (4). Linear regression modeled FRT inertial load-velocity relationships; multivariate regression integrated FRT load, velocity, and barbell %1RM (Figure 3).

### 2.6 Statistical analyses

Statistical analyses were performed using SPSS (v26.0, Chicago, USA) and R 4.3.2. Prior to data processing, normality (Shapiro–Wilk test) and homogeneity of variance (Levene’s test) were assessed. Data are presented as mean ± SD. Pearson’s correlation coefficient (r) quantified variable associations, while the coefficient of determination (R^2^) evaluated regression model fit. Linear and multivariate regression models were constructed with inertia (kg·m^2^) as the dependent variable and other factors as independent variables. Interpretation thresholds were defined as follows: r < 0.1 (negligible), 0.1–0.39 (weak), 0.4–0.69 (moderate), 0.7–0.89 (strong), and ≥0.9 (very strong) (29); R^2^ < 0.09 (negligible), 0.09–0.24 (low), 0.25–0.48 (moderate), 0.49–0.80 (high), and ≥0.81 (very high) (30). Statistical significance was set at *p* < 0.05, with non-significance defined as *p* > 0.05 (31). Test–retest reliability was assessed using absolute agreement intraclass correlation coefficients [ICC (2, 1)] between first and second FRT velocity measurements under identical inertial loads. Within-session reliability and coefficient of variation (CV) quantified relative and absolute reliability (95% CI). ICC thresholds were: <0.5 (poor), 0.5–0.75 (moderate), 0.75–0.9 (good), and >0.9 (excellent) (32). Boxplot interquartile ranges (IQR) were interpreted as: Q1 (25th percentile), Q3 (75th percentile), with larger IQR indicating greater data dispersion and smaller IQR reflecting central tendency.

## 3 Results

Table 1 presents the baseline characteristics of the included participants.

### 3.1 Linear comparison of load-velocity models between flywheel and barbell resistance training

All data were normally distributed. Linear regression models analyzed the relationship between flywheel inertia and velocity. The fitting results revealed a strong linear relationship (R^2^ = 0.78) with a strong negative correlation (r = −0.88). The regression equation was: y (velocity) = 0.965–0.609 × inertial load (kg·m^2^). Individual linear fitting results demonstrated consistently high linearity across all participants (see Supplementary Figures S1–S39). For barbell load-velocity analysis, linear regression showed a strong linear relationship (R^2^ = 0.74) with a strong negative correlation (r = −0.86). The regression equation was: y (velocity) = 1.346–0.006 × %1RM (kg) (Figures 4, 5).

**Figure 4:**
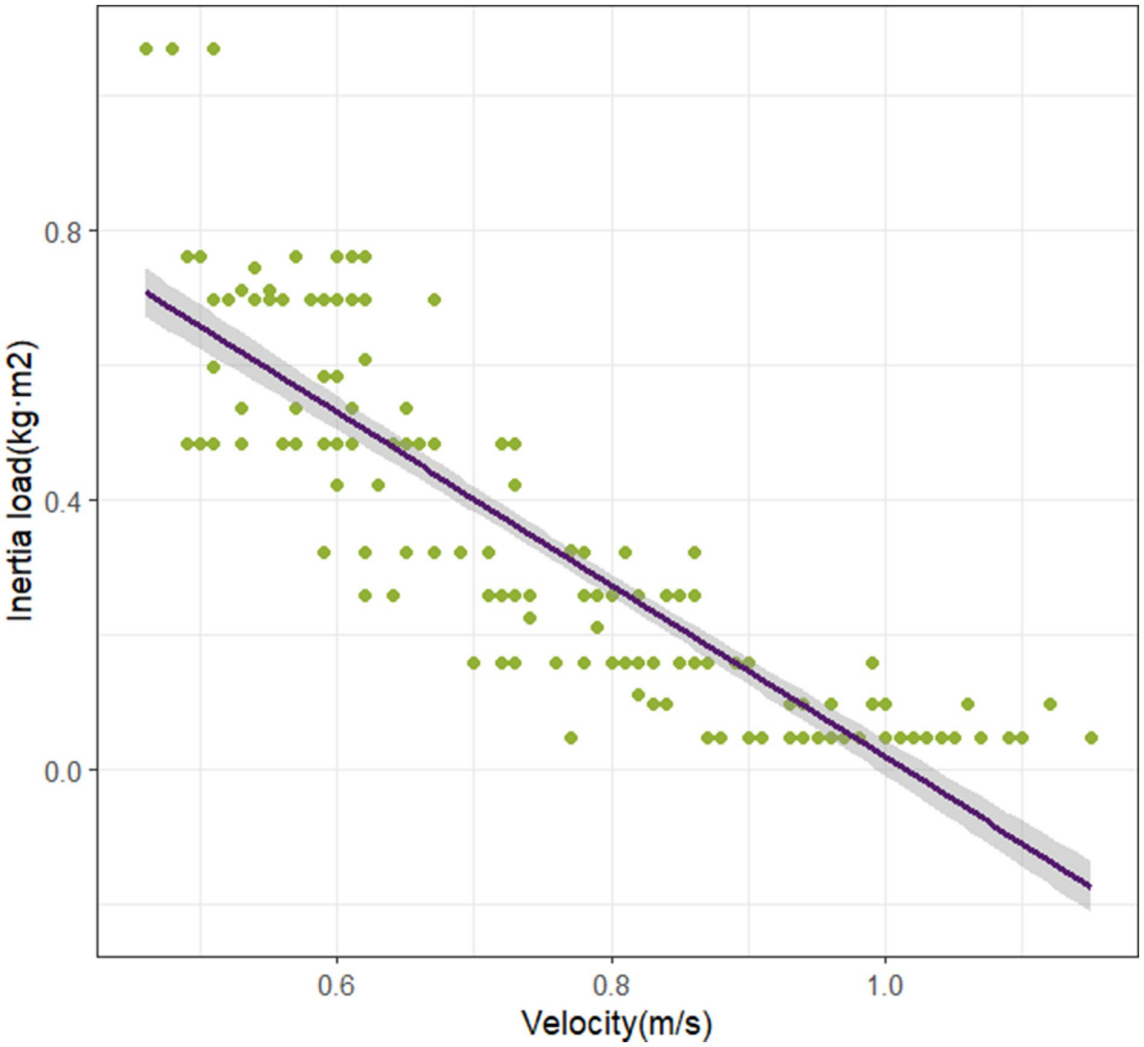
Inertia load-concentric velocity linear regression model.

**Figure 5:**
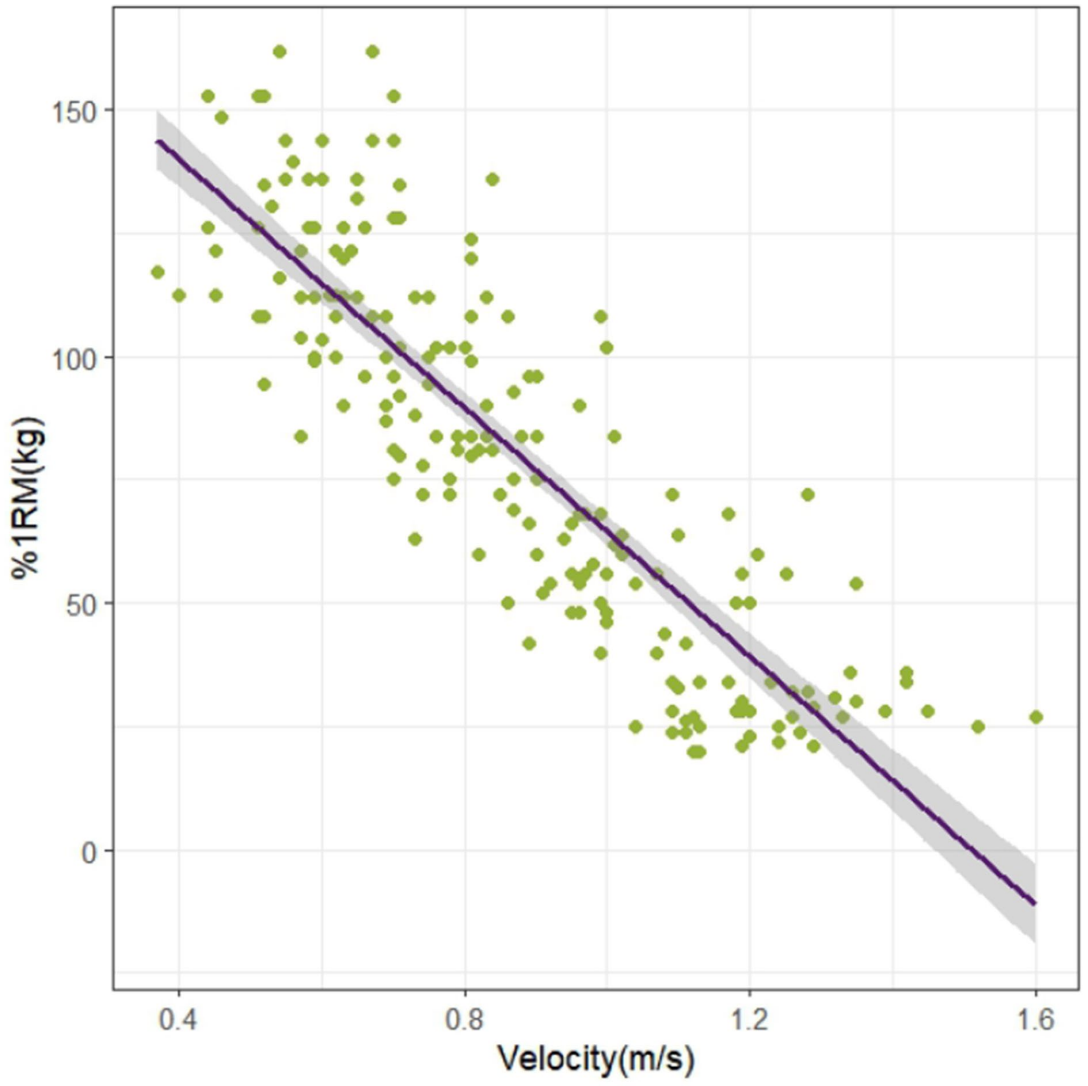
Barbell load-velocity linear regression model.

### 3.2 Boxplots and force-velocity profiles of participants’ different percentage inertial loads and velocities

**Figure 6:**
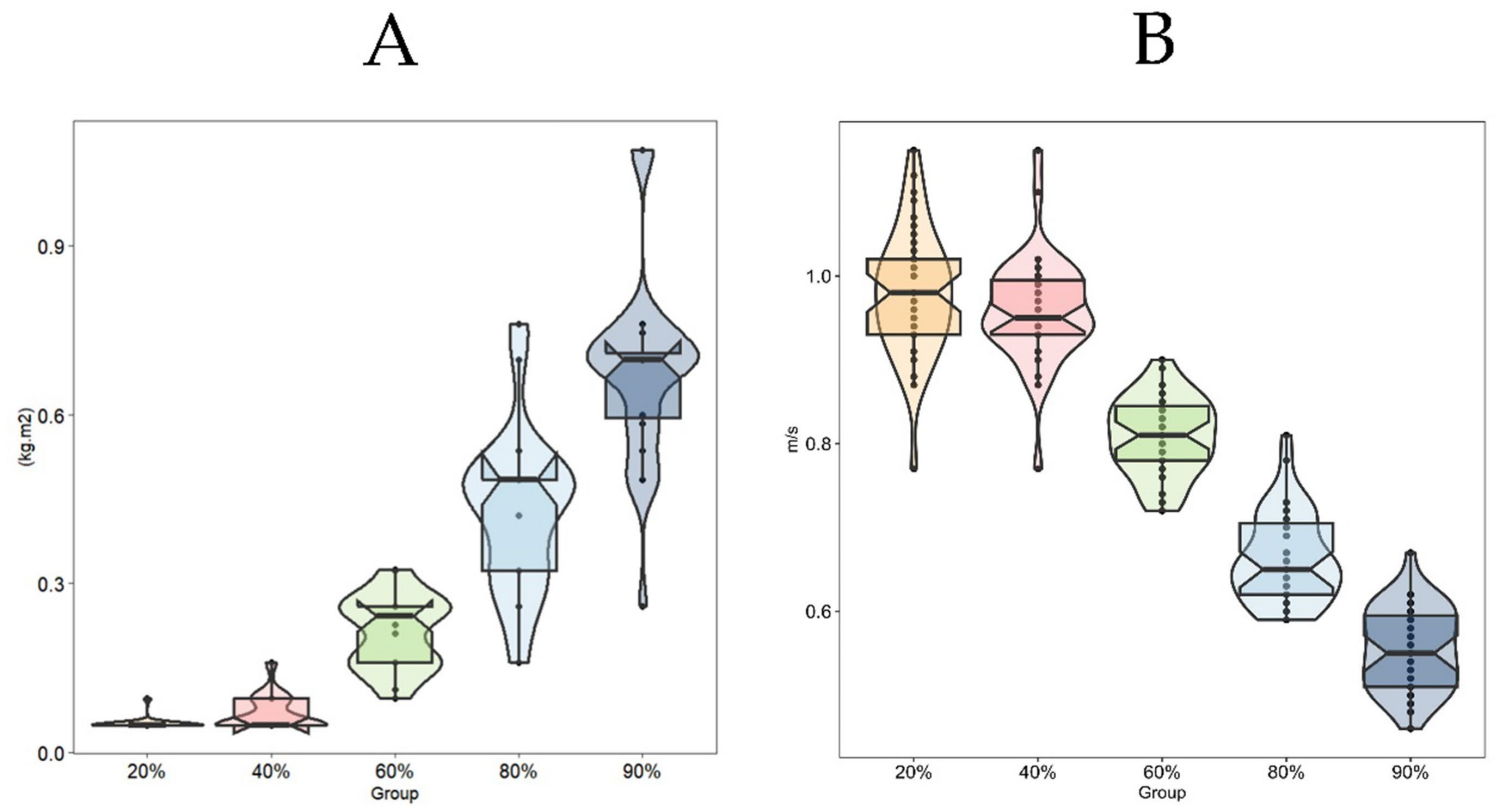
Inertia load distribution under different concentric percentages **(A)** and concentric velocity distribution under different concentric percentages **(B)**.

Figure 6 presents boxplot distributions of inertia (Figure 6A) and velocity (Figure 6B) for participants at different %1RM in the flywheel. It illustrates the distribution of load and velocity among participants at different %1RM (Table 2).

**Table 2:** Regression results of predictive indicators.

Variable	Coefficient	SD	*t*	*P*	r	R^2^	Adjusted R^2^
Velocity	−0.846	0.09	−9.235	0.00	0.90	0.81	0.81
%1RM	0.002	0.00	5.469	0.00			

%1RM, Weight as a percentage of the 1RM.

### 3.3 Constructing a multiple linear regression model with different indicators

The results show that the degree of fitting between inertia and velocity and different %1RM is quite high (R^2^ = 0.81) and there is a very strong correlation (r = 0.90). Regression equation y (inertia) = 0.769–0.846*v (velocity) + 0.002*kg (%1RM).

### 3.4 Flywheel test reliability assessment

The reliability analysis results indicated that the ICC for velocity across different loads all demonstrated moderate reliability, with low variability (Table 3).

**Table 3:** Flywheel load reliability assessment.

Variable	Mean ± SD	ICC (2, 1)	CV
		95%CI	
FRT 20%1RM Velocity	0.98 ± 0.07	0.64 (0.80–0.41)	0.07
FRT 40%1RM Velocity	0.96 ± 0.06	0.61 (0.78–0.37)	0.06
FRT 60%1RM Velocity	0.80 ± 0.05	0.53 (0.72–0.27)	0.06
FRT 80%1RM Velocity	0.66 ± 0.06	0.74 (0.86–0.55)	0.09
FRT 90%1RM Velocity	0.55 ± 0.05	0.60 (0.77–0.35)	0.09

## 4 Discussion

**Figure 7:**
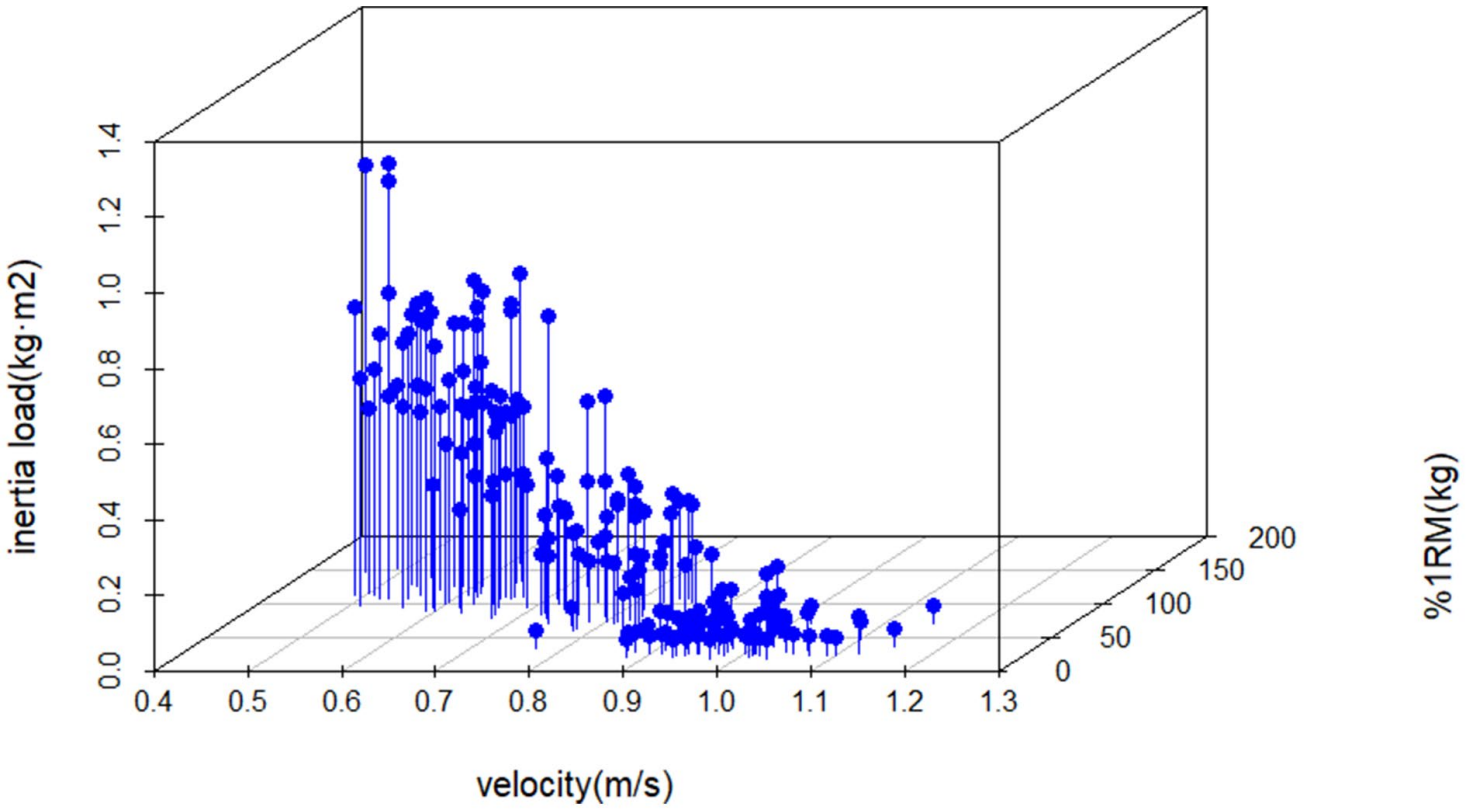
3D space multiple linear regression model.

This cross-sectional study systematically investigates the optimal load selection strategy for FRT based on established theoretical frameworks, constructing a FRT load parameter system through analysis of traditional barbell 1RM% load-velocity relationships. The research concurrently validates the effectiveness of velocity metrics in scientific monitoring of FRT. Results demonstrate a significant negative correlation in FRT load-velocity curves (r = −0.88), with regression model goodness-of-fit reaching R^2^ = 0.78 (Figure 4). The inertia-velocity-force multivariate regression model exhibits excellent goodness-of-fit (R^2^ = 0.81) and significant correlation (R = 0.90) (Figure 7). Multivariate regression models incorporating individualized velocity and strength parameters provide valid foundations for developing scientific FRT training protocols.

This study utilized the Gymaware system to synchronously capture concentric velocity data from both training modalities, corroborating findings from Martin-Rivera et al. (16) and McErlain-Naylor et al. (11). A pronounced inverse correlation was observed between inertial load and velocity (r = −0.88), aligning with the biomechanical principle that increased resistance loading induces velocity decrements (22, 33). Regression models revealed strong linear trends in both FRT and barbell resistance training (RT) (Figures 4, 5). However, FRT data exhibited homogeneous clustering across participants, a pattern consistent with the model characteristics described by Martin-Rivera et al. (16). The limited selection of inertial discs (s = 2, m = 1, l = 2, *p* = 2) constrained athletes’ load adjustments within a narrow range. Furthermore, FRT’s unique mechanical demands necessitate sustained maximal effort during the concentric phase (9, 34), coupled with dual resistance from inertial loading and kinetic energy conversion during the eccentric phase (7). Strength-dominant athletes require greater eccentric control force under identical loads and must overcome secondary inertial resistance at 90° squat depth (35). This dynamic load equilibrium may explain the observed homogeneous training responses across individuals. Our findings suggest that velocity-based unidimensional load monitoring may inadequately reflect inter-individual strength variability. We propose integrating individualized %1RM (kg) as a critical parameter for personalized FRT load prescription. The data distribution patterns provide practitioners with quantifiable references for individual differences, though their underlying mechanisms warrant further multidimensional investigation. Figure 7 demonstrates a multiple linear regression model for flywheel inertia, using barbell velocity and %1RM as dependent variables to derive corresponding inertia values. The advantage of this method lies in practitioners’ ability to calculate precise inertia values through regression equations by measuring easily accessible barbell data, given its high goodness of fit (R^2^ = 0.81). Additionally, since no prior studies have attempted to quantify the optimal loads for developing specific capacities through Flywheel Resistance Training (FRT), and insufficient evidence exists to directly identify these loads, this study adopted velocity zones from barbell training (36), as these zones have been validated as effective for targeting corresponding capacities. Previous studies reported that FRT should utilize velocity as a metric for monitoring load intensity (7, 11). However, to address inter-individual strength variations, different %1RM values were incorporated into the regression equation to ensure more accurate calculation of personalized inertia loads.

Personalized training prescriptions serve as an effective approach to optimize athletic performance while preventing injuries and overtraining (37), necessitating practitioners to center individualized athlete characteristics when developing sport-specific capacity enhancement strategies. This study established an evaluation framework accommodating diverse strength levels by integrating concentric velocity with corresponding %1RM (kg) load capacities. While low-inertia loads induce rightward shifts in the force-velocity curve, higher inertia may better stimulate upward displacement (11, 16). Although McErlain-Naylor et al. (11) proposed low inertia as optimal for individuals with “velocity deficits,” our results demonstrated that FRT velocities rarely reached 1.1–1.2 m/s at 20–40%1RM loads (Figures 6B, 7). Compared to velocity-based training (VBT) guidelines recommending 20–40%1RM loads at 1.0–1.3 m/s for speed-strength development (36), these findings suggest limited applicability of FRT for this objective. This limitation may stem from two FRT-specific mechanisms: firstly, rope length adjustments inherently restrict explosive force initiation; secondly, low-inertia loads reduce power output efficiency through constrained movement trajectories. Mechanistically, FRT demands maximal concentric effort (9, 34), with eccentric resistance arising from both inertial loads and kinetic energy transfer from prior concentric actions (7), requiring strength-dominant athletes to generate greater eccentric control forces under identical loads. At the 90° squat position, athletes must overcome persistent flywheel inertia (35), a dynamic resistance absent in traditional barbell squats where explosive jumps enable full force release at lighter loads (38). These inherent constraints highlight the need to incorporate individualized %1RM (kg) as a critical parameter for FRT load prescription, as velocity-based monitoring alone may inadequately reflect inter-individual strength variability.

FRT has demonstrated efficacy in developing chronic muscular adaptations (12, 13, 35, 39) and elicits significant acute effects through Post-activation Performance Enhancement (PAPE) responses (40, 41). Previous investigations employing fixed loading plates for parameter selection introduce substantial methodological errors when comparing training modalities, primarily due to intermodal load disparities. Our methodology enabled velocity-matched conversion of traditional barbell percentages to flywheel equivalents, ensuring comparable concentric resistance while generating transient eccentric overload through the device’s unique movement pattern (9). This coupled concentric-eccentric modality potentially enhances athletic performance to a greater extent with flywheel implementation.

**Figure 8:**
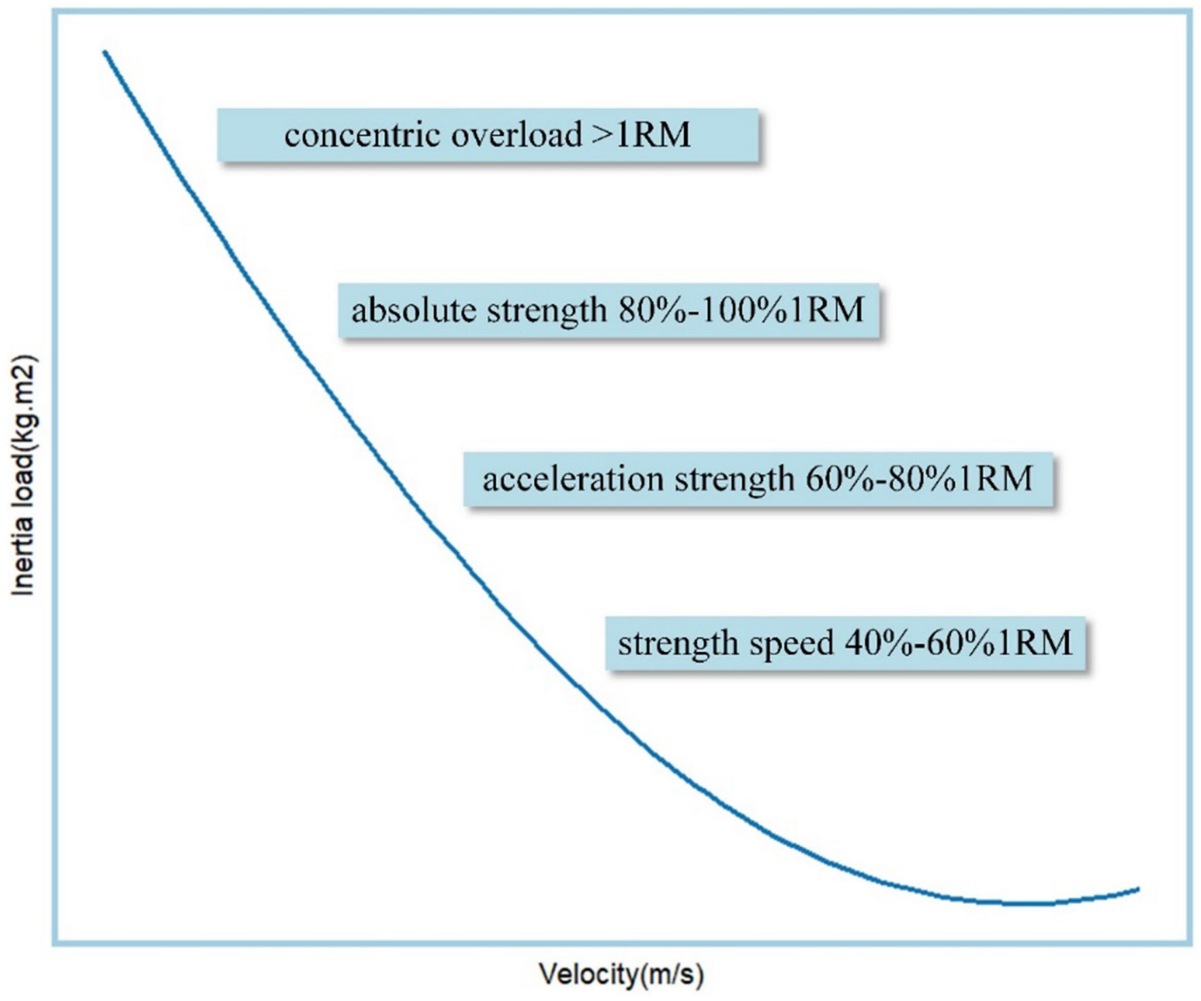
Barbell velocity-based FRT corresponding development capability.

Figure 8 presents the zones corresponding to the development of distinct capabilities in Flywheel Resistance Training (FRT), derived from this study’s findings based on the “Velocity-Based Training” prescription. Signore et al. (36) replaced the traditional 1RM-based training model with velocity zones in “Velocity-Based Training,” modifying the traditional force-velocity curve by dividing it into speed ranges where each interval corresponds to specific force adaptations, as each strength type or athletic quality operates optimally within defined velocity ranges (36). Compared to traditional 1RM methods, velocity-monitored training demonstrates greater efficacy in achieving significant performance improvements (42). This study identified FRT-specific loads by analyzing concentric velocities under different barbell percentage loads. Aligning with Signore et al. (36) force-velocity curve, FRT effectively develops athletes’ absolute strength (80–100%1RM or higher), explosive power (60–80%1RM), and strength-speed (40–60%1RM). The force-velocity curve describes the neuromuscular system’s mechanical force production during multi-joint movements (21), and FRT’s inertia-induced velocity reduction aligns with Hill et al. (22) classical force-velocity relationship, suggesting that the proposed load-selection method may optimize training outcomes due to FRT’s ability to maximize muscle activation throughout concentric and partial eccentric phases (41, 43). Studies indicate that accelerating concentrically after the eccentric phase enhances elastic energy utilization and propulsion velocity, matching sport-specific force patterns (44). However, FRT’s effectiveness in the strength-speed zone (<40%1RM) exhibits strong individual variability: most participants used “S” load plates, with few utilizing “S + S” configurations, a phenomenon requiring larger-scale investigations.

## 5 Conclusion

This study provides new evidence for quantifying the effects of flywheel load on developing different athletic capacities. FRT demonstrates a highly linear relationship between inertia load and velocity. By fitting regression equations of inertia load with velocity and force, it is possible to personalize the inertia load for male athletes with varying strength levels. The research indicates the following load ranges for different strength qualities in FRT: 40–60%1RM (strength-speed), 60–80%1RM (power), and 80–100%1RM or even exceeding 1RM (maximal strength). Additionally, FRT is not suitable for developing speed-strength, but whether it can yield better benefits in periodized long-term training remains to be tested.

## 6 Limitations

(a) The limited selection of experimental subjects may restrict the generalizability of the research findings. Future studies should expand the sample size and include more populations, such as the general population and female groups. (b) The study used kinematic indicators for measurement and did not include kinetic indicators. Therefore, assumptions such as homogeneity in movement patterns and techniques during athlete testing must be considered. (c) The study is cross-sectional in design and has not been validated in practice. Future researchers should attempt periodic experimental studies to verify the effectiveness of the regression equations. (d) The inertial velocity in the study was difficult to reach higher levels. Future research should continue to explore the reasons for this phenomenon, whether it is due to limitations of the training equipment, measurement technology, or movement execution methods.

## 7 Practical applications

Coaches should select the velocity to be used and the weight (%1RM) that can be overcome at the relative intensity according to the athlete’s training goal, and calculate the corresponding inertia through the regression equation to avoid under loading or overtraining. At the same time, it is necessary to know where the FRT corresponds to the force-velocity graph for the development of different abilities.

## Data Availability

The original contributions presented in the study are included in the article/Supplementary material, further inquiries can be directed to the corresponding author.
