# Supplementary material for: An investigation of the load-velocity relationship between flywheel eccentric and barbell training methods
Source: Front Public Health. 2025 May 30;13:1579291. doi: 10.3389/fpubh.2025.1579291 (PMC12162595; doi:10.3389/fpubh.2025.1579291)
Supplement: Supplementary file 2 [file Data_Sheet_2.docx]

1 Individual Linear Regression Models for Participants (Flywheel Load vs. Velocity)

#1 Fengrui Zhang

Regression Equation: Inertia=1.3766−1.4154⋅Velocity

R^2^=0.90


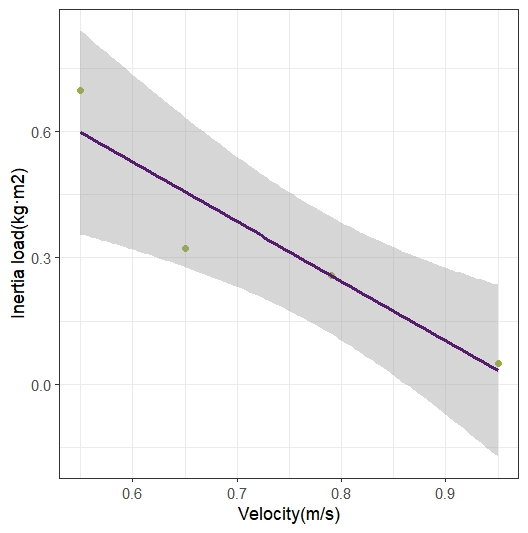


Figure S1 Linear Fitting Model for Flywheel Load and Velocity

#2 Xikai Luo

Regression Equation: Inertia=1.3001−1.2336⋅Velocity

R^2^=0.93


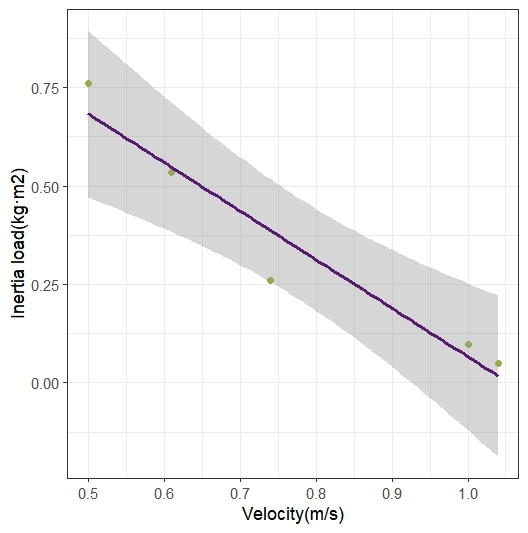


Figure S2 Linear Fitting Model for Flywheel Load and Velocity

#3 Yingxian Nie

Regression Equation: Inertia= 1.4510 -1.4872⋅Velocity

R^2^=0.91


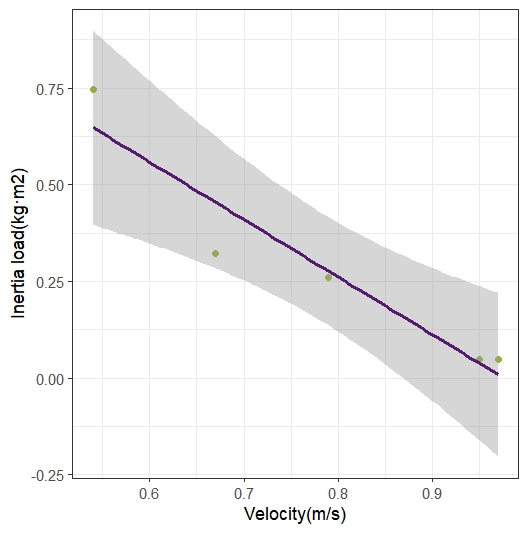


Figure S3 Linear Fitting Model for Flywheel Load and Velocity

#4 Shengzhang Zhu

Regression Equation: Inertia=2.0100-2.0723⋅Velocity

R^2^=0.97


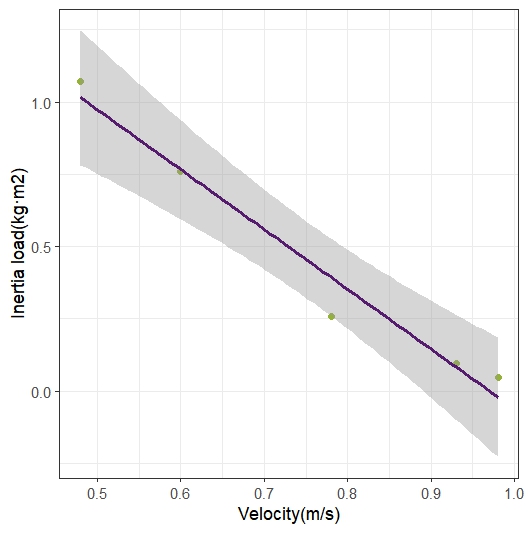


Figure S4 Linear Fitting Model for Flywheel Load and Velocity

#5 Xin Yao

Regression Equation: Inertia=1.2070−1.1042⋅Velocity

R^2^=0.92


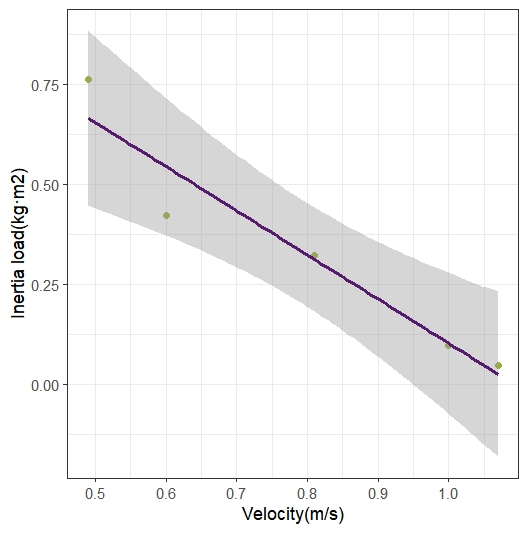


Figure S5 Linear Fitting Model for Flywheel Load and Velocity

#6 Yong Liu

Regression Equation: Inertia=1.1579−1.1611⋅Velocity

R^2^=0.95


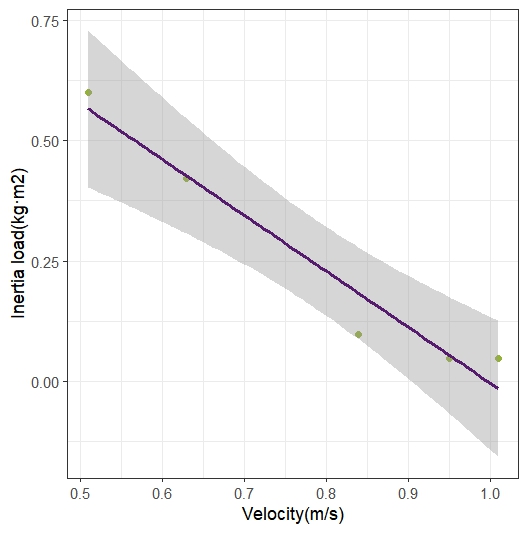


Figure S6 Linear Fitting Model for Flywheel Load and Velocity

#7 Zeyang Liu

Regression Equation: Inertia=1.0231−0.9593⋅Velocity

R^2^=0.90


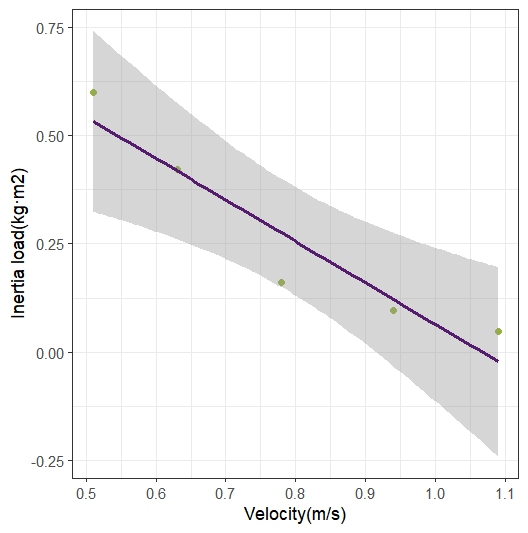


Figure S7 Linear Fitting Model for Flywheel Load and Velocity

#8 Baolin Xu

Regression Equation: Inertia=1.8097−1.6185⋅Velocity

R^2^=0.96


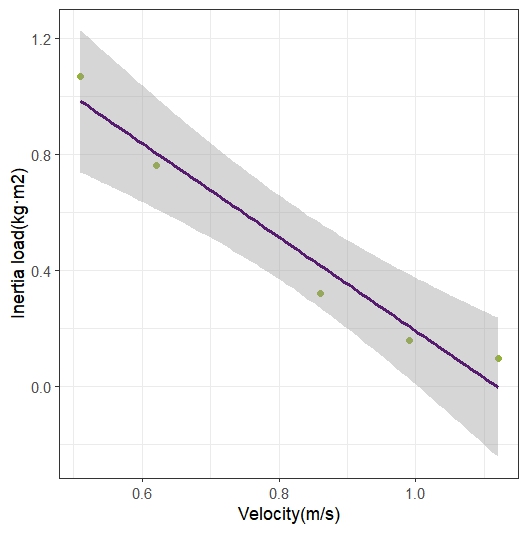


Figure S8 Linear Fitting Model for Flywheel Load and Velocity

#9 Juxin Liang

Regression Equation: Inertia=1.5879−1.4799⋅Velocity

R^2^=0.88


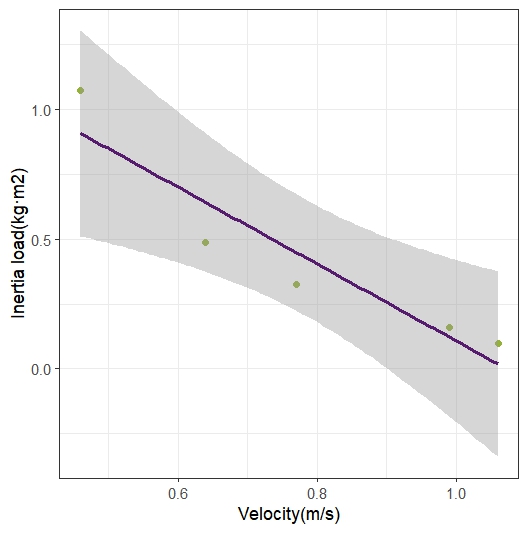


Figure S9 Linear Fitting Model for Flywheel Load and Velocity

#10 Tiancheng Ma

Regression Equation: Inertia=0.73663−0.78120⋅Velocity

R^2^=0.99


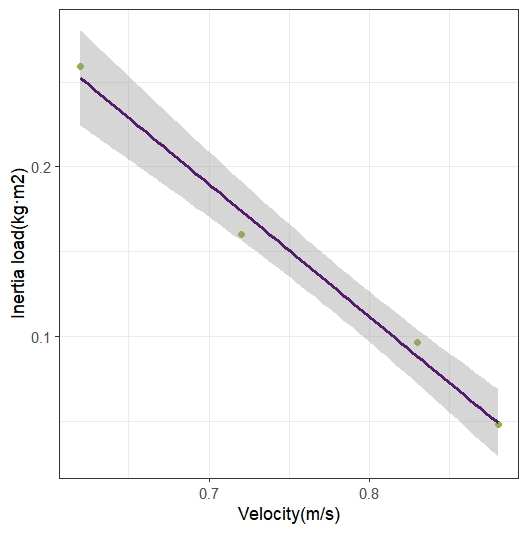


Figure S10 Linear Fitting Model for Flywheel Load and Velocity

#11 Haoguo Xu

Regression Equation: Inertia=1.0788−1.0740⋅Velocity

R^2^=0.92


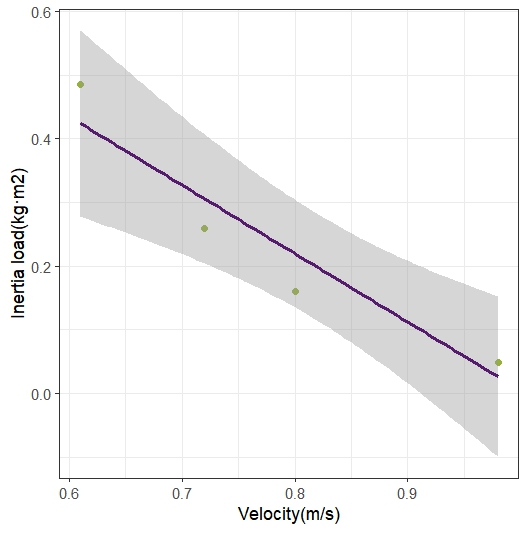


Figure S11 Linear Fitting Model for Flywheel Load and Velocity

#12 Minjian Liao

Regression Equation: Inertia=1.6582−1.6827⋅Velocity

R^2^=0.96


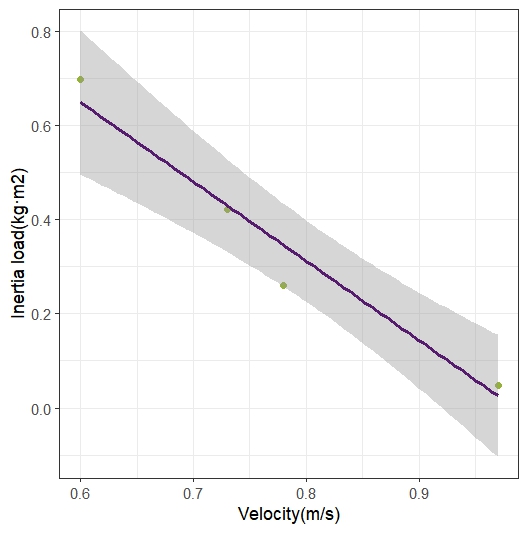


Figure S12 Linear Fitting Model for Flywheel Load and Velocity

#13 Xuewu Li

Regression Equation: Inertia=2.08837−2.23365⋅Velocity

R^2^=0.99


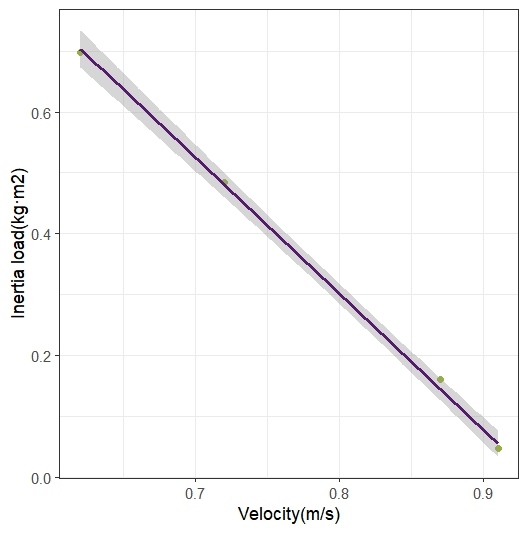


Figure S13 Linear Fitting Model for Flywheel Load and Velocity

#14 Duo Wang

Regression Equation: Inertia=1.35105−1.28837⋅Velocity

R^2^=0.99


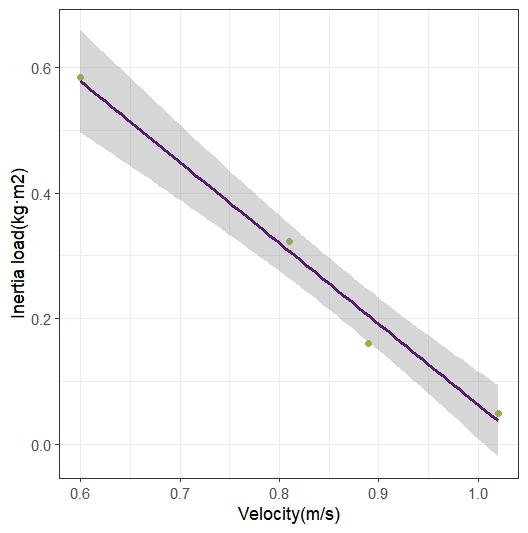


Figure S14 Linear Fitting Model for Flywheel Load and Velocity

#15 Quanqiang Long

Regression Equation: Inertia=1.1457−1.1016⋅Velocity

R^2^=0.90


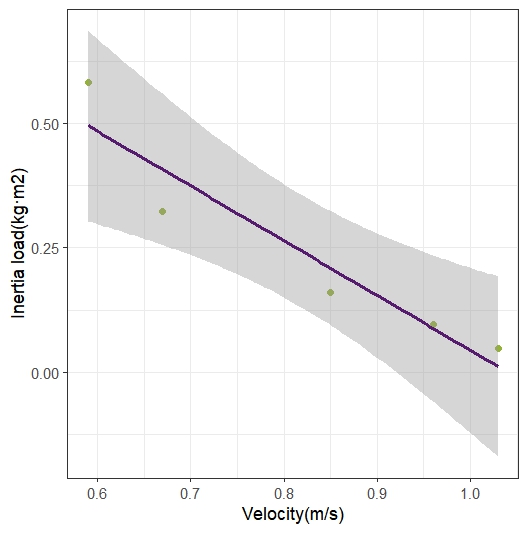


Figure S15 Linear Fitting Model for Flywheel Load and Velocity

#16 Sirui Luo

Regression Equation: Inertia=1.3975−1.3170⋅Velocity

R^2^=0.95


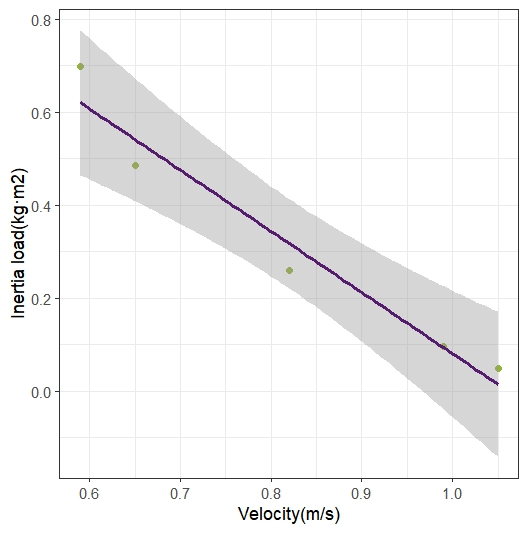


Figure S16 Linear Fitting Model for Flywheel Load and Velocity

#17 Kaizhi Yao

Regression Equation: Inertia=2.1513−2.3394⋅Velocity

R^2^=0.87


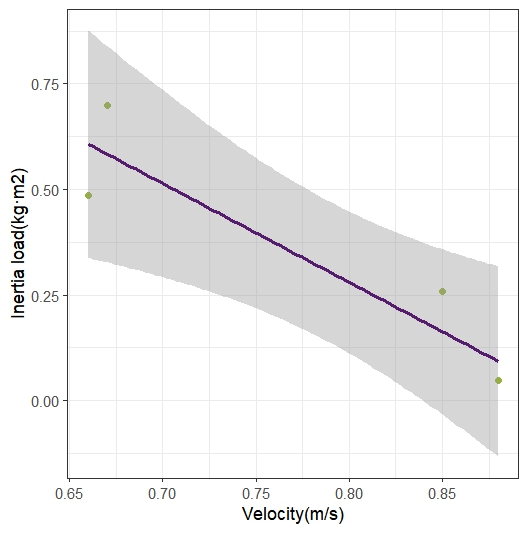


Figure S17 Linear Fitting Model for Flywheel Load and Velocity

#18 Zhihao Li

Regression Equation: Inertia=1.767062−1.753847⋅Velocity

R^2^=1.00


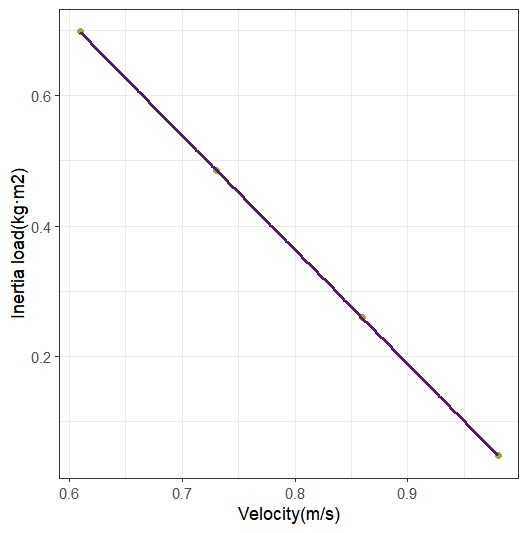


Figure S18 Linear Fitting Model for Flywheel Load and Velocity

#19 Bingbo Yuan

Regression Equation: Inertia=1.25746−1.18997⋅Velocity

R^2^=0.98


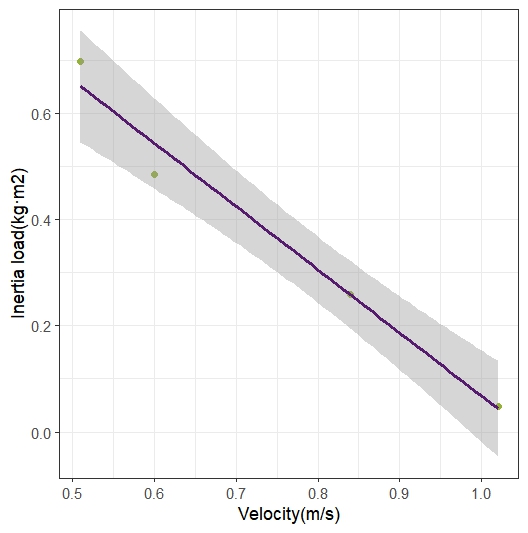


Figure S19 Linear Fitting Model for Flywheel Load and Velocity

#20 Tianxing Ye

Regression Equation: Inertia=1.25746−1.18997⋅Velocity

R^2^=0.81


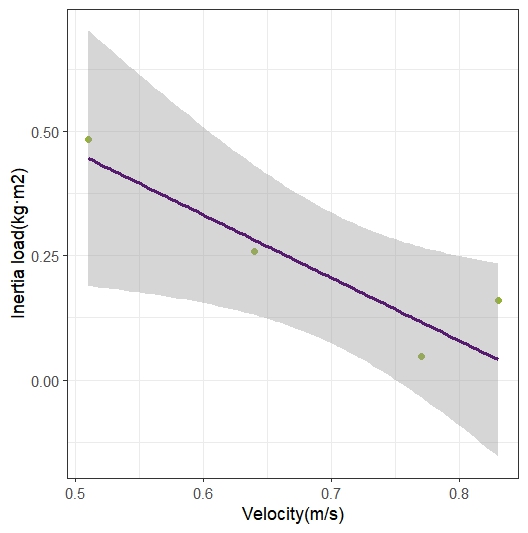


Figure S20 Linear Fitting Model for Flywheel Load and Velocity

#21 Weiwen Xu

Regression Equation: Inertia=1.2216−1.2547⋅Velocity

R^2^=0.86


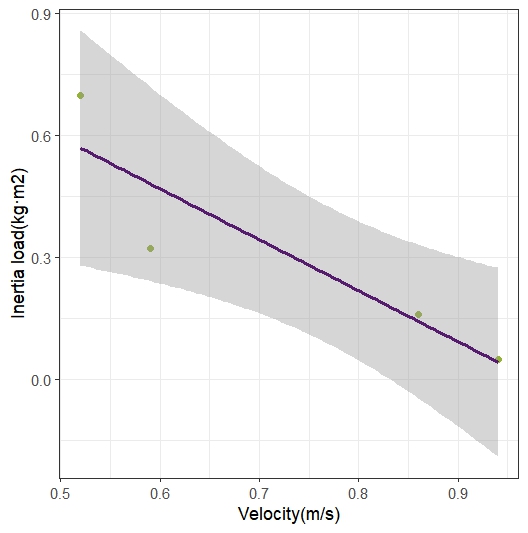


Figure S21 Linear Fitting Model for Flywheel Load and Velocity

#22 Jialin Hou

Regression Equation: Inertia=1.5728−1.7140⋅Velocity

R^2^=0.94


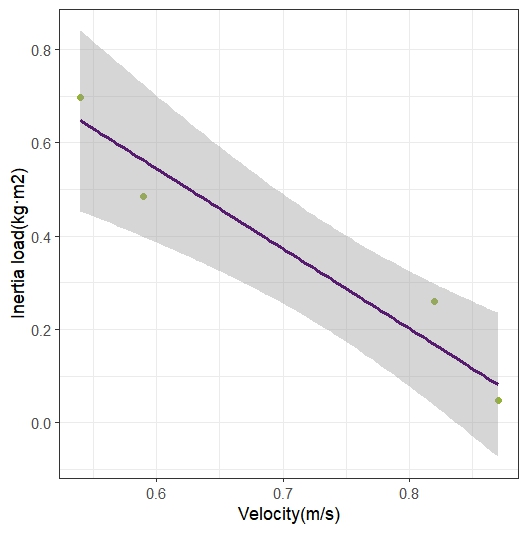


Figure S22 Linear Fitting Model for Flywheel Load and Velocity

#23 Bin Zeng

Regression Equation: Inertia=1.7890−1.8235⋅Velocity

R^2^=0.98


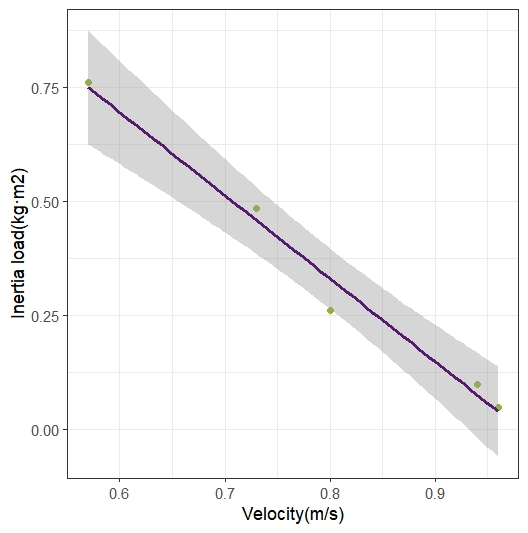


Figure S23 Linear Fitting Model for Flywheel Load and Velocity

#24 Shuai Wang

Regression Equation: Inertia=0.8754−0.7683⋅Velocity

R^2^=0.93


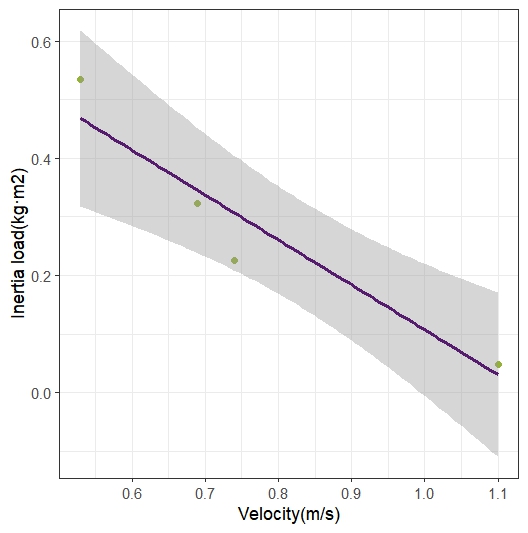


Figure S24 Linear Fitting Model for Flywheel Load and Velocity

#25 Junhui Tu

Regression Equation: Inertia=1.7354−1.8944⋅Velocity

R^2^=0.93


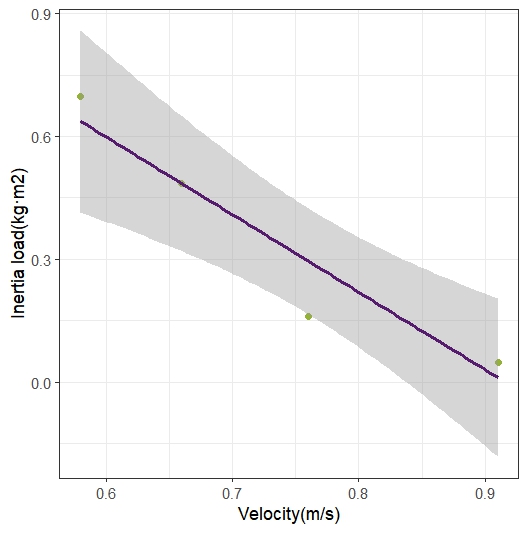


Figure S25 Linear Fitting Model for Flywheel Load and Velocity

#26 Shengbo Wang

Regression Equation: Inertia=1.4966−1.5023⋅Velocity

R^2^=0.92


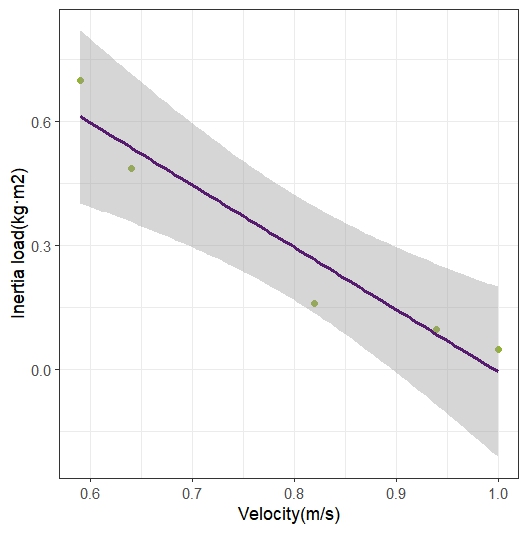


Figure S26 Linear Fitting Model for Flywheel Load and Velocity

#27 Senquan Peng

Regression Equation: Inertia=1.6872−1.7539⋅Velocity

R^2^=0.94


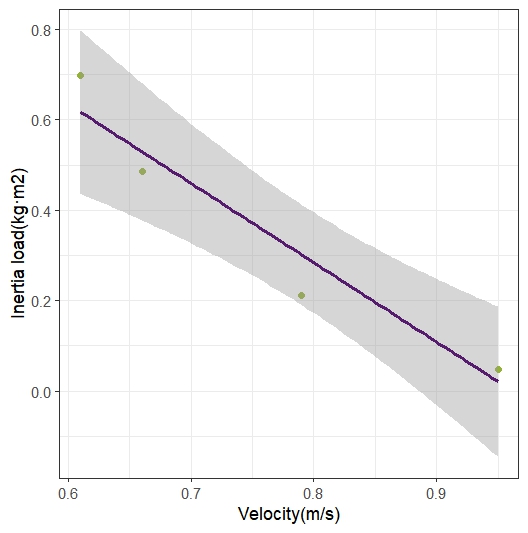


Figure S27 Linear Fitting Model for Flywheel Load and Velocity

#28 Kai Hu

Regression Equation: Inertia=1.65424−1.73951⋅Velocity

R^2^=0.99


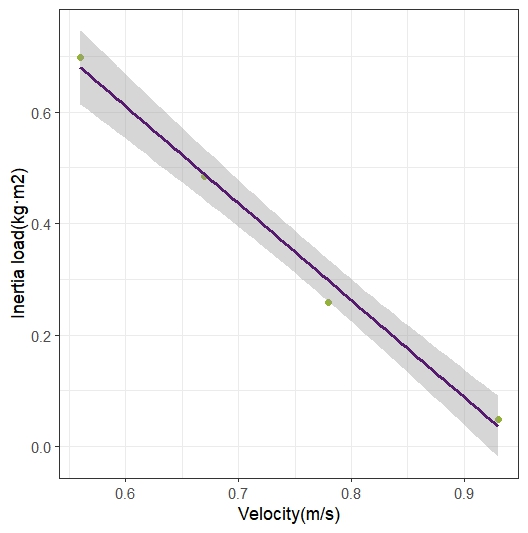


Figure S28 Linear Fitting Model for Flywheel Load and Velocity

#29 Debiao Cai

Regression Equation: Inertia=1.6169−1.7124⋅Velocity

R^2^=0.98


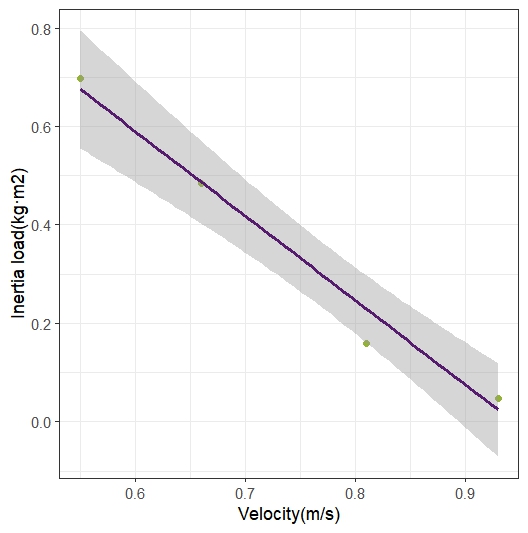


Figure S29 Linear Fitting Model for Flywheel Load and Velocity

#30 Bohao Liu

Regression Equation: Inertia=0.9680−1.0436⋅Velocity

R^2^=0.94


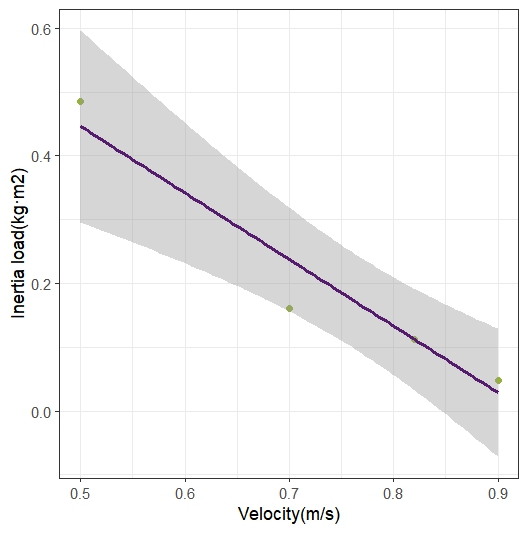


Figure S30 Linear Fitting Model for Flywheel Load and Velocity

#31 Tao Yu

Regression Equation: Inertia=1.4662−1.2592⋅Velocity

R^2^=0.95


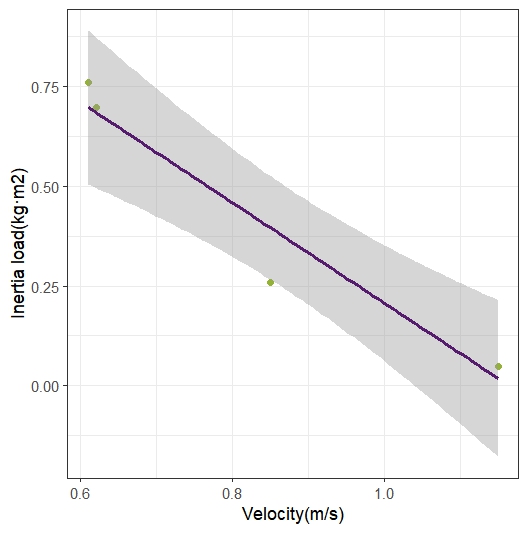


Figure S31 Linear Fitting Model for Flywheel Load and Velocity

#32 Hui Han

Regression Equation: Inertia=0.95483−0.98766⋅Velocity

R^2^=0.98


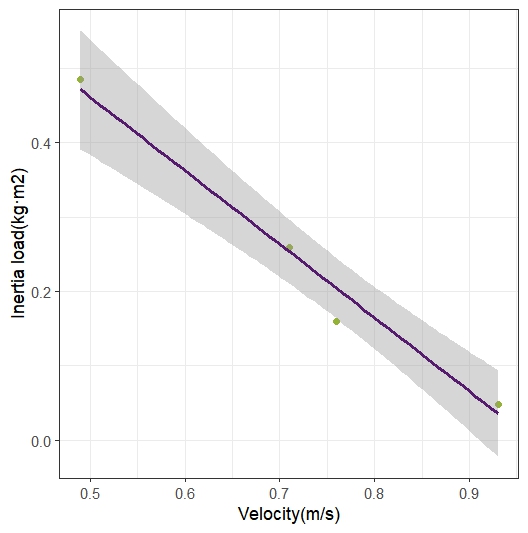


Figure S32 Linear Fitting Model for Flywheel Load and Velocity

#33 Chenchan Lin

Regression Equation: Inertia=1.4504−1.4553⋅Velocity

R^2^=0.93


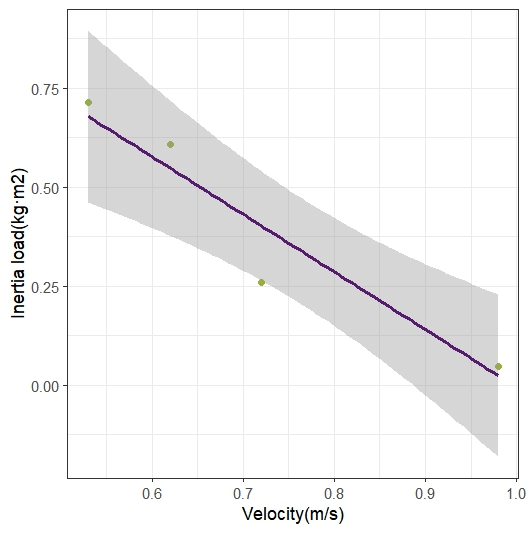


Figure S33 Linear Fitting Model for Flywheel Load and Velocity

#34 Junhong Li

Regression Equation: Inertia=1.16118−1.18829⋅Velocity

R^2^=1.00


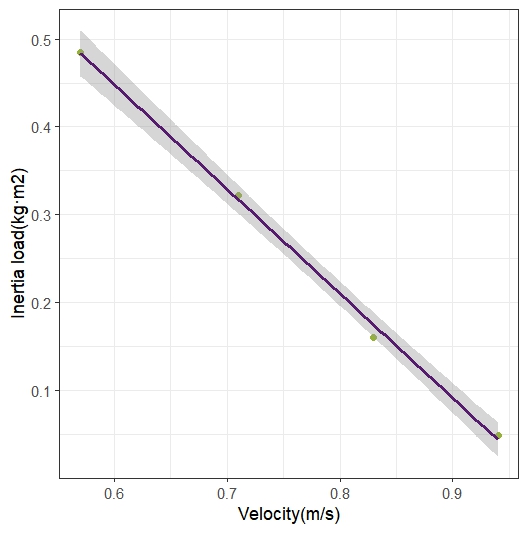


Figure S34 Linear Fitting Model for Flywheel Load and Velocity

#35 Jianteng Fan

Regression Equation: Inertia=1.27555−1.33002⋅Velocity

R^2^=0.99


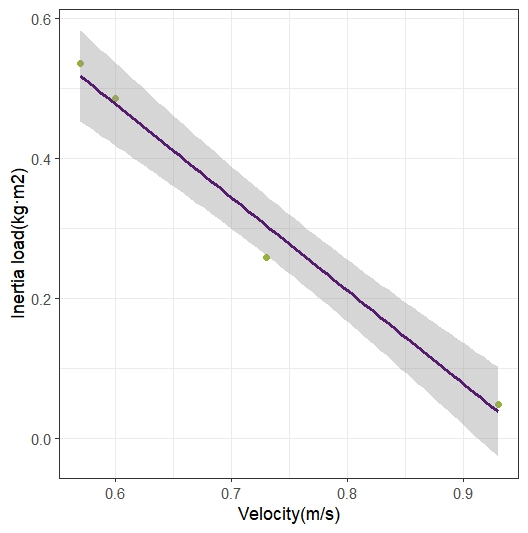


Figure S35 Linear Fitting Model for Flywheel Load and Velocity

#36 Xuewen He

Regression Equation: Inertia=0.8429−0.8077⋅Velocity

R^2^=0.90


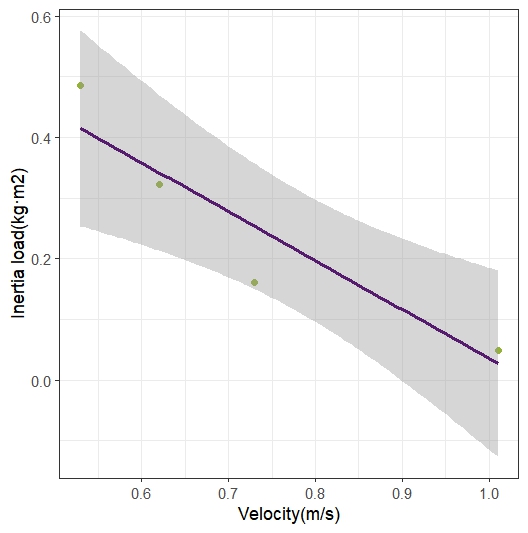


Figure S36 Linear Fitting Model for Flywheel Load and Velocity

#37 Rongfeng Zhou

Regression Equation: Inertia=0.9252−0.8881⋅Velocity

R^2^=0.93


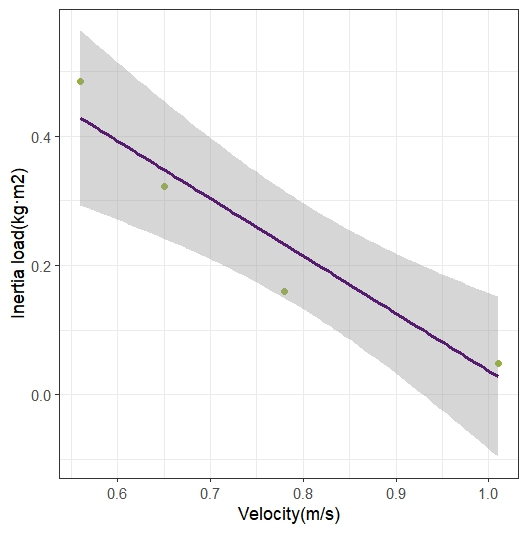


Figure S37 Linear Fitting Model for Flywheel Load and Velocity

#38 Lin Xie

Regression Equation: Inertia=1.4960−1.4751⋅Velocity

R^2^=0.98


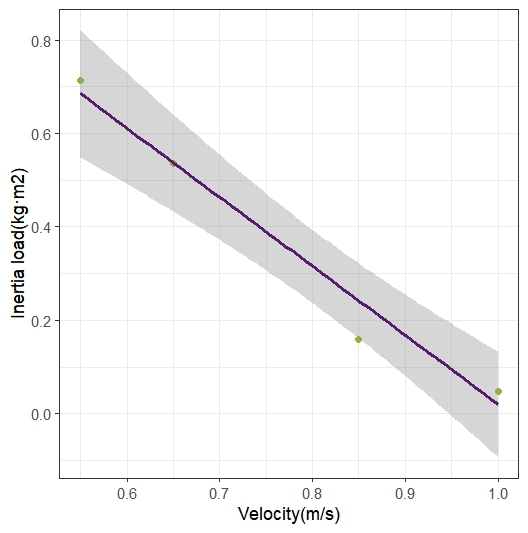


Figure S38 Linear Fitting Model for Flywheel Load and Velocity

#39 Wuwen Peng

Regression Equation: Inertia=1.17045−1.11081⋅Velocity

R^2^=1.00


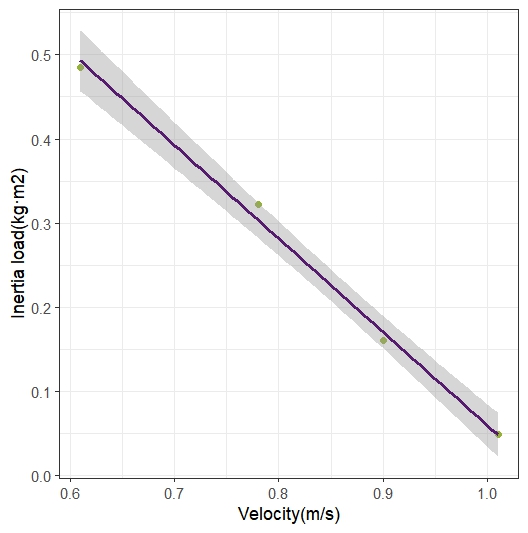


Figure S39 Linear Fitting Model for Flywheel Load and Velocity
